# Culturable bacteria and fungi in *Ixodes*, *Dermacentor*, *Amblyomma* and *Ornithodoros* ticks

**DOI:** 10.1051/parasite/2025013

**Published:** 2025-03-25

**Authors:** Marjorie Bruley, Célia Pasternicki, Noor Fattar, Julien Amoros, Maxime Duhayon, Karen McCoy, Olivier Duron

**Affiliations:** 1 MIVEGEC (Maladies Infectieuses et Vecteurs : Ecologie, Génétique, Evolution et Contrôle), University of Montpellier (UM), Centre National de la Recherche Scientifique (CNRS), Institut pour la Recherche et le Développement (IRD) 34394 Montpellier France; 2 ASTRE, CIRAD, INRAE, University of Montpellier 34398 Montpellier France

**Keywords:** Ticks, Microbiome, Fungi, Bacteria

## Abstract

Ticks are ectoparasites harboring complex microbial communities, typically dominated by nutritional symbionts that produce B vitamins and sometimes including pathogens affecting human and animal health. However, ticks also host a variety of commensal microbes whose diversity remains poorly documented. In this study, we isolated and identified culturable bacteria and fungi associated with various tick species from the genera *Ixodes*, *Dermacentor*, *Amblyomma*, and *Ornithodoros*, collected from their natural habitats or hosts in France and French Guiana. A total of 111 bacterial and 27 fungal isolates were obtained which were then identified using both molecular and morphological approaches. Substantial fungal diversity was observed in a few ticks, whereas culturable bacteria displayed a broader distribution and diversity across tick species. Interestingly, the diversity of culturable bacteria and fungi revealed a microbiome structure that reflected the ecological niches of the tick host, indicating habitat-specific microbial associations and a potential ecological role in tick biology. The isolation of common gut bacteria of other arthropods, as well as the isolation of a viable entomopathogenic fungus, underscores the potential influence of these microbes on tick biology.

## Abstract (fr)

Les tiques sont des ectoparasites abritant des communautés microbiennes complexes, généralement dominées par des symbiotes nutritionnels qui produisent des vitamines B et parfois incluant des pathogènes affectant la santé humaine et animale. Cependant, les tiques hébergent également une variété de microbes commensaux dont la diversité reste mal documentée. Dans cette étude, nous avons isolé et identifié des bactéries et champignons cultivables associés à diverses espèces de tiques des genres *Ixodes*, *Dermacentor*, *Amblyomma* et *Ornithodoros*, collectées dans leurs habitats naturels ou hôtes en France et en Guyane française. Au total, 111 isolats bactériens et 27 isolats fongiques ont été obtenus, qui ont ensuite été identifiés à l’aide d’approches moléculaires et morphologiques. Une diversité fongique substantielle a été observée chez quelques tiques, tandis que les bactéries cultivables présentaient une distribution et une diversité plus larges parmi les espèces de tiques. Il est intéressant de noter que la diversité des bactéries et des champignons cultivables a révélé une structure du microbiome qui reflétait les niches écologiques de l’hôte tique, indiquant des associations microbiennes spécifiques à l’habitat et un rôle écologique potentiel dans la biologie des tiques. L’isolement de bactéries intestinales communes d’autres arthropodes, ainsi que l’isolement d’un champignon entomopathogène viable, soulignent l’influence potentielle de ces microbes sur la biologie des tiques.

## Introduction

Ticks are among the most significant vectors of pathogens that affect both humans and animals [16, 37, 45]. Major tick-borne pathogens include bacteria such as *Borrelia burgdorferi* sensu lato, the causative agent of Lyme disease, as well as viruses, protozoa, and filarial nematodes [16, 37, 45]. This list of tick-borne pathogens is continually expanding, with new microorganisms being regularly identified [5, 9, 10, 12, 40, 49]. However, ticks also harbor a variety of non-pathogenic microbes, including commensal and mutualistic species [6, 8, 20, 45, 53]. Ticks feed exclusively on blood throughout all stages of their life cycle, and their internal microbiome is both unique and complex. For example, extracellular bacteria residing in the tick gut can modulate the tick’s immune responses, thereby influencing their resistance to pathogens [1, 44]. Maternally inherited intracellular bacteria likewise play a critical role in tick development and survival [2, 21, 29, 62]. As blood is nutritionally imbalanced, ticks rely on narrow associations with B vitamin-providing intracellular symbionts to synthesize these essential nutrients [18, 20]. In addition to their internal microbes, ticks also harbor a highly diverse external (cuticular) microbiota formed by a broad range of microorganisms [4]. The influence of this external microbial community on tick biology, however, remains largely unexplored.

Tick-borne pathogens and internal microbes are often highly specialized to the tick’s physiological environment, and are either difficult to culture or completely unculturable [6, 8, 19, 20, 43]. As a result, these microbes are primarily studied using DNA-based molecular techniques, with microscopy playing a secondary role [6, 8, 19, 20, 43]. Importantly, the advent of next-generation sequencing technologies has dramatically increased the number of bacteria identified as part of the tick microbiome [6, 8, 45, 53]. The composition of whole tick microbiota now appears to be highly variable and context-dependent, with the local environment shaping the presence, abundance, and diversity of bacterial communities [15, 43, 45, 53, 58]. The external microbes of the tick microbiome can result from contamination by the skin microbiome of their vertebrate hosts during blood feeding and by environmental microbes from the soil, plants, or vertebrate nests and burrows during the “off-host” periods [15, 43, 45, 53, 58]. Some of these microbes may exist as long-term commensals on ticks with minimal impact, while others can act as opportunistic pathogens affecting tick survival [6, 30]. However, while some bacteria identified through molecular surveys may genuinely associate with ticks, others may be DNA environmental contaminants representing organisms that do not associate or survive on the tick body surface [58].

In this study, we focus on the living, and culturable, microbes that either reside on the surface of ticks or inhabit their internal environments. To this end, we isolated bacteria and fungi from six tick species using a standardized approach in aerobic media. We first collected field specimens of four tick species in France (*Ixodes ricinus*, *Ixodes frontalis*, *Dermacentor marginatus*, and *Ornithodoros* (*Alectorobius*) *maritimus*) and of two species in French Guiana (*Amblyomma cajennense* and *Dermacentor nitens*). All of these species belong to the Ixodidae family (hard ticks), except for *O. maritimus*, which belongs to the Argasidae family (soft ticks). The castor bean tick, *I. ricinus*, and the Cayenne tick, *A. cajennense*, are major vectors of tick-borne pathogens infecting humans in Europe and South America, respectively [3, 50, 51]. The ornate sheep tick, *D. marginatus*, and the tropical horse tick, *D. nitens*, are natural vectors of protozoan parasites that infect wild and domestic animals, especially horses [3, 50]. The other two tick species exhibit host specialization, with *I. frontalis* primarily feeding on passerine birds and *O. maritimus* predominantly parasitizing seabirds [50]. We identified culturable bacteria and fungi in these collected ticks through molecular typing and analyzed the partition of their taxonomic diversity among tick species. While previous studies have examined the diversity of culturable bacteria in *I. ricinus* in some European countries (Belgium [54], Hungary [25], Czech Republic [55], Poland [46, 59]), no studies have been performed in France. No data are currently available for the other five tick species.

## Materials and methods

### Ethics

The French Ministry of Ecological Transition has validated the collection and use of tick samples from French Guiana under the reference TREL19028117S/156, in accordance with the provisions of the Nagoya Protocol on Access and Benefit-Sharing (ABS).

### Tick collection

Ticks were sampled from their natural habitats or vertebrate hosts in France and French Guiana (Table 1). A collection of 47 field specimens from six tick species, including *O. maritimus* (*n* = 5), *I. ricinus* (*n* = 4), *I. frontalis* (*n* = 5), *D. marginatus* (*n* = 6), *D. nitens* (*n* = 10), and *A. cajennense* (*n* = 17) were obtained. Specimens of *I. ricinus*, *I. frontalis*, and *A. cajennense* were questing (ungorged) ticks collected from vegetation using the flagging method. Specimens of the other three species were engorged ticks: *O. maritimus* were specifically collected from gull nests, while *D. marginatus* and *D. nitens* were taken from horses. Nymphs and adults were gathered with sterile forceps and individually stored in sterile tubes. Larvae were not sampled. Identifications of the collected ticks were made using morphological identification keys for all species [27, 50], and molecular typing for *Amblyomma* nymphs [3].

**Table 1:** List and origin of tick specimens examined in this study.

Tick species	Number (nymphs/♂/♀)	Sampling locality
**Argasidae (soft ticks)**		
*Ornithodoros maritimus* (Vermeil & Marguet, 1967)	5 (0/0/0/5)	Carteau, France, 2024 (seabird nests)
**Ixodidae (hard ticks)**		
*Amblyomma cajennense* (Fabricius, 1787)	17 (0/17/0/0)	Montsinéry-Tonnegrande, French Guiana, 2023 (vegetation)
*Ixodes frontalis* (Panzer, 1798)	5 (0/5/0/0)	Saint-Julien-de-la-Nef, France, 2024 (vegetation)
*Ixodes ricinus* (Linnaeus, 1758)	4 (0/0/2/2)	Bourgogne, France, 2024 (vegetation)
*Dermacentor marginatus* (Sulzer, 1776)	6 (0/0/1/5)	Pompignan, France, 2024 (horses)
*Dermacentor nitens* (Neumann, 1897)	10 (0/0/2/8)	Tonate-Macouria, French Guiana, 2023 (horses)

### Microbial culture and isolation

For culturing, each whole tick body was longitudinally cut into two equal parts using a sterile blade. One half was stored at −80 °C for later use, while the other half was used for microbial culture. Tick half samples used for culturing were individually crushed using a pestle, then homogenized in sterile tubes containing 1000 μL of brain heart infusion (BHI) growth medium (PanReac AppliChem, ITW Reagents, Chicago, IL, USA). The homogenized tick mixtures were then incubated overnight at room temperature (20 °C ± 1) in aerobic conditions. After incubation, 100 μL of each homogenized mixture were spread using a rake on Columbia agar plates (Condalab, Madrid, Spain) containing 5% sheep blood, then incubated at room temperature in aerobic conditions. After 48-h of incubation, the morphological properties of microbial colonies were used to select colonies for further assays: shape, size, surface structure, color, transparency, and texture. Pure cultures of each selected colony were grown on fresh agar plates. Each pure culture was photographed using a Scan 300 (Interscience) camera. To ensure representative sampling and minimize taxonomic bias, in instances of a tick specimen yielding two visually identical microbial colonies, only one was retained for further analysis. Specifically, one pure culture per morphological type and per tick specimen was thus selected. Control samples obtained from growth media and culture tools revealed no contamination by external microbes.

### Molecular identification of bacterial and fungal isolates

DNA was extracted for each pure colony using a DNeasy Blood and Tissue Kit (QIAGEN, Hilden, Germany), following manufacturer’s instructions. For bacterial colonies, a 1500 bp fragment of 16S rDNA was amplified by standard PCR and sequenced using the universal primers 8F (5′-GAGTTTGATCMTGGCTCAG-3′) [24] and 1507R (5′-ACGGNTACCTTGTTACRACTT-3′) [41]. For fungal colonies, a 400 bp fragment of 18S rDNA was amplified by standard PCR and sequenced using the universal primers NSF4/18 (5′-CTGGTTGATYCTGCCAGT-3′) and NSR399/19 (5′-TCTCAGGCTCCYTCTCCGG-3′) [31]. PCR assays were performed in a total volume of 25 μL and contained 8 mM of each dNTP (Thermo Scientific, Waltham, MA, USA), 10 mM of MgCl2 (Thermo Scientific), 7.5 μM of each of the internal primers, 2.5 μL of 10× PCR buffer (Thermo Scientific), and 1.25 U of Taq DNA polymerase (Thermo Scientific). All PCR amplifications were performed as follows: initial denaturation at 94 °C for 2 min, 35 cycles of denaturation (94 °C, 30 s), annealing (52 °C, 30 s), extension (72 °C, 2 min and 1 min, for 16S rDNA and 18S rDNA, respectively), and a final extension at 72 °C for 5 min. Each PCR assay included positive controls, featuring DNA from *Pseudomonas* sp. (for 16S rDNA) and *Pilaira* sp. (for 18S rDNA), as well as a negative control with water. Following visualization via electrophoresis in 1.5% agarose gel, positive PCR products were sequenced using Sanger technology (Eurofins, Luxembourg). Sequence chromatograms were cleaned with Chromas Lite (http://www.technelysium.com.au/chromas_lite.html), and alignments were performed using ClustalW, implemented in the MEGA software package (https://www.megasoftware.net/). The Basic Local Alignment Search Tool (BLAST; https://blast.ncbi.nlm.nih.gov/blast/Blast.cgi) was used to find available sequences showing nucleotide similarities. These sequences were then compared with bacterial and fungal isolates to examine sequence identity in nucleotide alignments for 16S rRNA and 18S rRNA.

### Molecular phylogenetics

Phylogenetic analyses were based on sequence alignments of 16S rRNA and 18S rRNA gene fragments. The Gblocks program with default parameters was used to obtain non-ambiguous sequence alignments [14]. Tree-based phylogenetic analyses were performed using the maximum-likelihood (ML) method using the MEGA software package (https://www.megasoftware.net/). The evolutionary models that best fit the sequence data were determined using the Akaike information criterion. Clade robustness was assessed by bootstrap analysis using 1000 replicates.

### Statistics

All statistical analyses were conducted using R (http://www.r-project.org/). To assess the correlation between sampling effort and the number of bacterial and fungal isolates, Spearman’s rank correlation test was used. The difference in the number of bacterial and fungal isolates between tick species was tested using Fisher’s exact tests. To examine the variation in the number of bacterial and fungal isolates between species from different regions (France *vs.* French Guiana), a PERMANOVA test was performed, utilizing the vegan package and the Adonis function.

### Data availability

Nucleotide sequences were deposited in the GenBank nucleotide database (16S rRNA: PQ683868–PQ683978; 18S rRNA: PQ669579–PQ669905).

## Results

### Isolation and typing of tick culturable microbes

**Figure 1:**
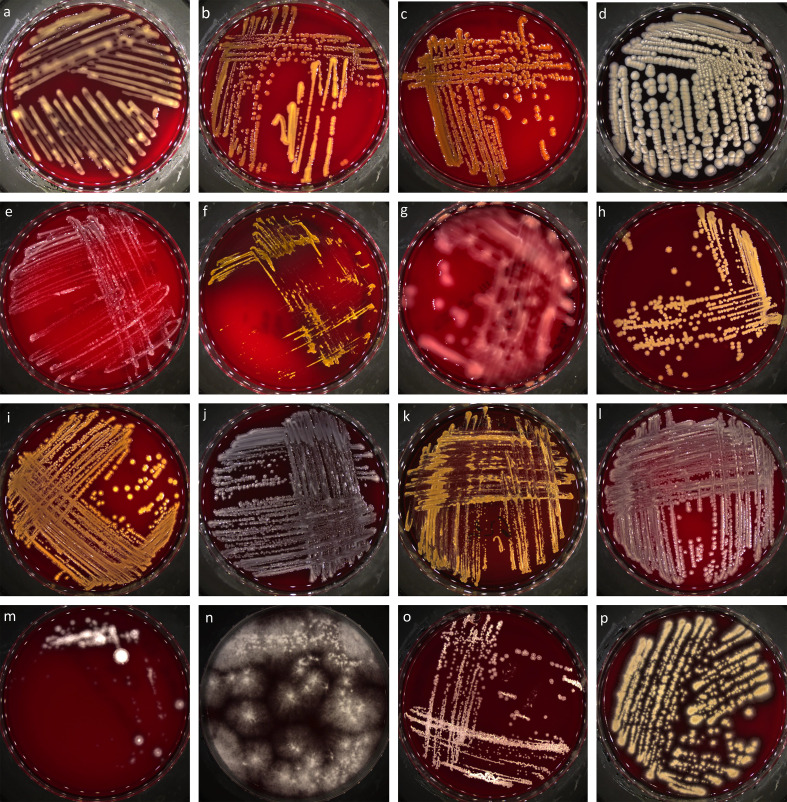
Illustrative examples of bacterial (**a-l**) and fungal (**m-p**) isolates obtained from ticks. (**a**) *Pantoea* sp. isolate c26a (*A. cajennense*), (**b**) *Curtobacterium* sp. isolate c22d (*A. cajennense*), (**c**) *Chryseobacterium* sp. isolate c38b (*A. cajennense*), (**d**) *Bacillus* sp. isolate c11b (*D. nitens*), (**e**) *Halomonas* sp. isolate c54b (*O. maritimus*), (**f**) *Sphingomonas* sp. isolate c62i (*I. frontalis*), (**g**) *Pseudomonas* sp. isolate c47c (*I. ricinus*), (**h**) *Staphylococcus* sp. isolate c51a (*D. nitens*), (**i**) *Exiguobacterium* sp. isolate c47b (*I. ricinus*), (**j**) *Klebsiella* sp. isolate c4b (*D. nitens*), (**k**) *Rhodococcus* sp. isolate c62h (*I. frontalis*), (**l**) *Sphingobacterium* sp. isolate c47d (*I. ricinus*), (**m**) Hypocreales isolate c61a (*I. frontalis*), (**n**) Hypocreales isolate c2c (*D. nitens*), (**o**) Ustilaginales isolate c22c (*A. cajennense*), (**p**) Capnodiales isolate c27a (*A. cajennense*).

A total of 138 isolates, including 111 bacterial isolates and 27 fungal isolates, were obtained from the 47 field-collected ticks (Fig. 1, Tables S1 and S2). Culturable bacteria were consistently isolated from all tick specimens, with a range of 1–9 isolates per specimen and 9–32 isolates per tick species. In contrast, fungi were isolated from only 16 tick specimens (34%), with each positive specimen yielding between 1 and 4 fungal isolates. Fungi were isolated from *I. frontalis*, *A. cajennense*, and *D. nitens*, with the number of fungal isolates per species ranging from 3 to 15, while no fungus was isolated from *O. maritimus*, *I. ricinus*, and *D. marginatus* specimens. A greatest number of bacteria and fungi were isolated from *A. cajennense* (32 and 15, respectively, from 17 specimens) compared to other tick species, whereas the lowest number of isolates were obtained from *O. maritimus* (9 and 0, respectively, from 5 specimens). The number of isolates per tick species was not correlated with the screening effort, i.e., the number of specimens examined per tick species (Spearman’s rank correlation, *n* = 6, *r*_s_ = 0.57, *p* = 0.23).

16S rDNA gene sequencing of the 111 bacterial isolates led to the characterization of 78 distinct sequences (70.46–99.93% pairwise nucleotide identities), including 3 in *O. maritimus* (for 9 isolates), 7 in *I. ricinus* (12 isolates), 15 in *I. frontalis* (21 isolates), 5 in *D. marginatus* (10 isolates), 20 in *D. nitens* (27 isolates), and 29 in *A. cajennense* (32 isolates). Remarkably, the same 16S rDNA sequence is often identified across different isolates derived from distinct specimens of the same tick species, suggesting the presence of a single bacterial strain. For instance, all four *I. ricinus* specimens produced isolates with 16S rDNA sequences that were 100% identical (isolates c47b, c48a, c49a, and c50a), while these sequences were not found in any other tick species. Similarly, four *D. marginatus* specimens yielded isolates with 16S rDNA sequences that were also 100% identical (isolates c41, c42, c44, and c46b). Likewise, four *O. maritimus* specimens produced isolates with 16S rDNA sequences that were 100% identical to one another (isolates c52b, c53b, c54a, and c55a) but distinct from those associated with *D. marginatus*. Notably, none of the bacterial 16S rDNA sequences were shared between tick species, with the exception of one sequence that was found in both *I. ricinus* (isolates 47c and 49b) and *D. marginatus* (isolate 46a). The 18S rRNA gene fragment sequences of the 27 fungal isolates revealed the presence of 16 distinct sequences (77.62%–99.72% pairwise nucleotide identities), including 7 in *I. frontalis* (for 9 isolates), 2 in *D. nitens* (3 isolates), and 7 in *A. cajennense* (15 isolates). As with bacteria, the same 18S rDNA sequence can often be detected across different isolates obtained from distinct specimens of the same tick species, suggesting the presence of a single fungal strain. A notable example involves seven specimens of *A. cajennense*, which produced isolates with 18S rDNA sequences that were 100% identical (isolates c22c, c23c, c24c, c27b, c27c, c35a, c37b, and c38c, with c27b and c27 being derived from the same tick specimen). These sequences were unique to fungal isolates of *A. cajennense* and were not identified in any other tick species. Importantly, none of the fungal 18S rDNA sequences were shared between different tick species.

### Taxonomic assignation of the culturable tick microbes

**Figure 2:**
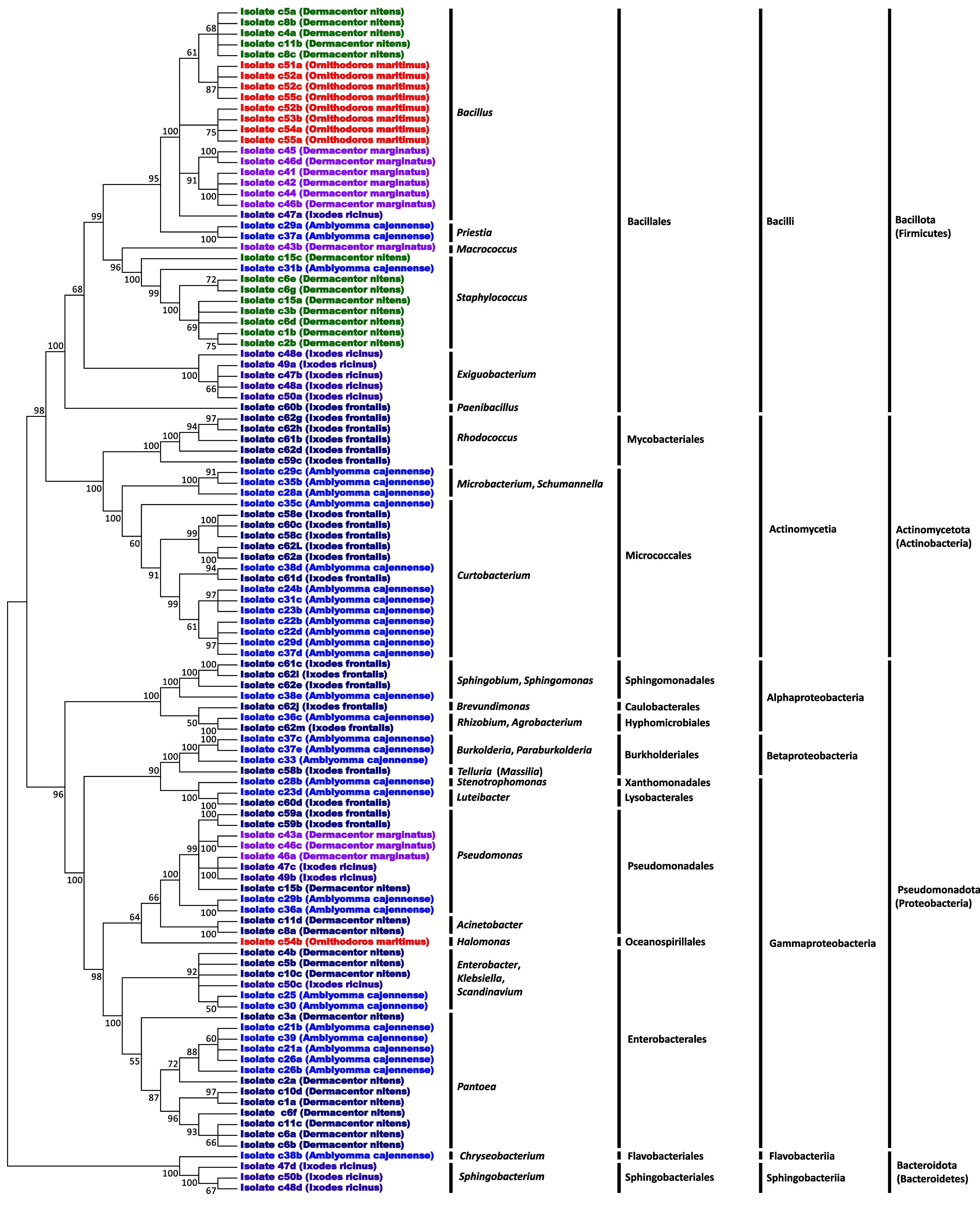
Cladograms depicting the 50% majority-rule consensus of bacterial phylogenetic trees constructed using maximum-likelihood (ML) estimations based on 16S rDNA gene sequences (1410 unambiguously aligned bp; best-fit approximation for the evolutionary model: GTR + G+I).

Most isolated culturable bacteria were from the Pseudomonadota (Proteobacteria) phylum (46 of 111 isolates), mainly from the *Pantoea* (13 isolates) and *Pseudomonas* (10 isolates) genera (Fig. 2 and Table S1). Other genera of Pseudomonadota were more rarely isolated and included *Burkholderia*, *Paraburkholderia*, *Rhizobium*, *Agrobacterium*, *Acinetobacter*, *Sphingobium*, *Sphingomonas*, *Brevundimonas*, *Telluria* (*Massilia*), *Stenotrophomonas*, *Luteibacter*, *Halomonas*, *Enterobacter*, *Klebsiella*, and *Scandinavium*. Most other isolated culturable bacteria were from the Bacillota (Firmicutes) phylum (38 of 111 isolates), mainly from the *Bacillus* (20 isolates), *Staphylococcus* (9 isolates), and *Exiguobacterium* (5 isolates) genera. The remaining isolated genera of Bacillota were *Priestia*, *Macrococcus* and *Paenibacillus*. Bacteria of the Actinomycetota (Actinobacteria) phylum were also isolated (23 of 111 isolates) and belonged to the *Curtobacterium* (14 isolates), *Rhodococcus* (5 isolates) and *Microbacterium*/*Shumannella* (4 isolates) genera. Less common bacteria were from the Bacteroidota (Bacteroidetes) phylum (4 isolates) with the *Chryseobacterium* (1 isolate) and *Sphingobacterium* (3 isolates) genera. These bacterial isolates exhibited high pairwise nucleotide identity either with environmental bacteria commonly found on vegetation, in the rhizosphere, and in soil, or with bacteria associated with arthropods (and, in some cases, specifically their midgut), but none were closely related to strains identified as human pathogens (Table S1).

**Figure 3:**
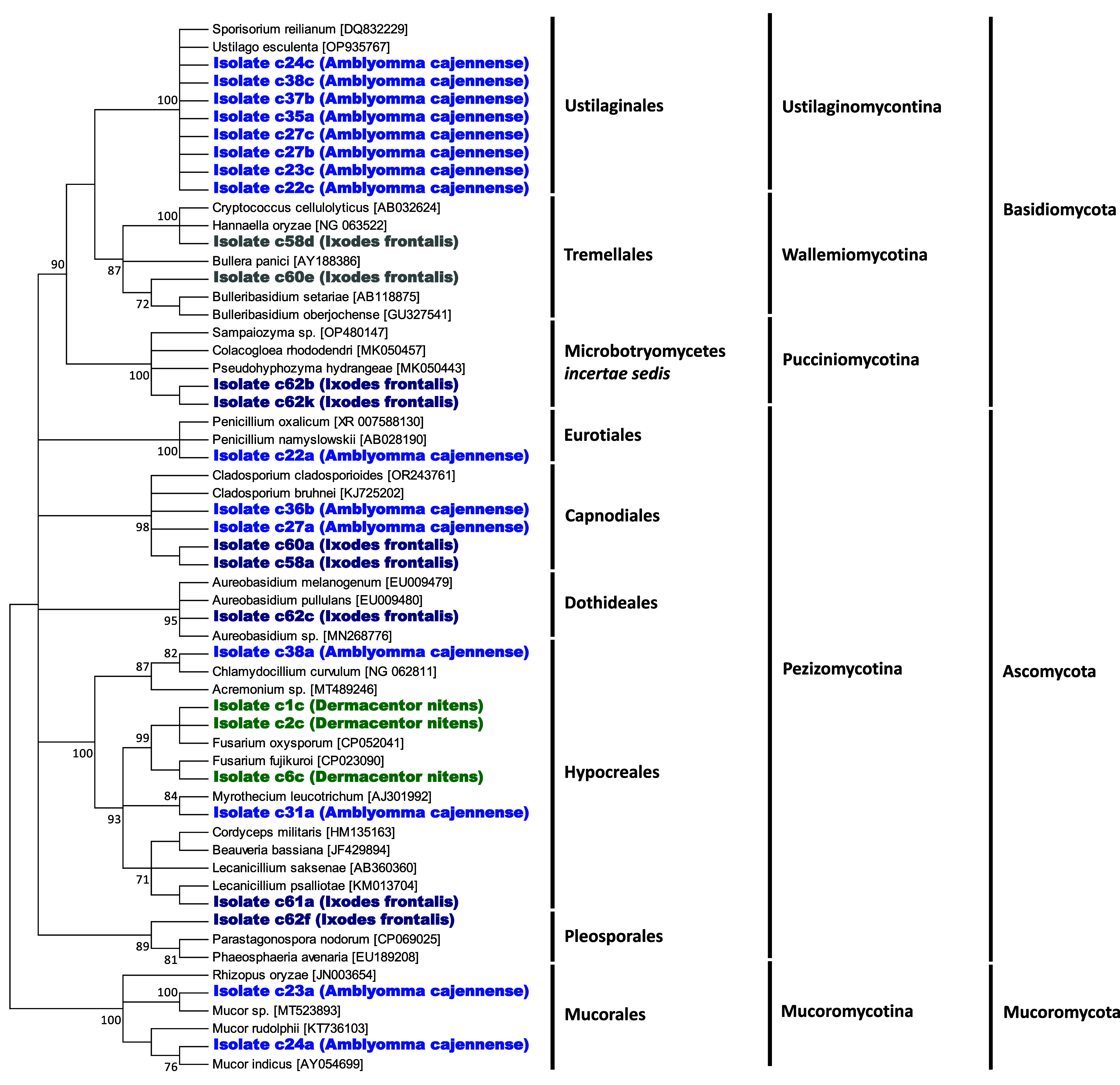
Cladograms depicting the 50% majority-rule consensus of fungal phylogenetic trees constructed using maximum-likelihood (ML) estimations based on 16S rDNA gene sequences (358 unambiguously aligned bp; best-fit approximation for the evolutionary model: HKY + G).

The 27 fungal isolates were categorized into three major divisions: Ascomycota (13 isolates), Basidiomycota (12 isolates), and Mucoromycota (2 isolates). Although the 18S rRNA gene sequences lacked sufficient polymorphism to definitively classify the isolates at the species or genus level, pairwise nucleotide identities and ML phylogenetic analyses enabled their grouping into nine distinct orders (Fig. 3 and Table S2). Within Ascomycota, 6 isolates belonged to the order Hypocreales, 4 to Capnodiales, and 1 to each order Pleosporales, Dothideales and Eurotiales. Interestingly, the Hypocreales isolate c61a, obtained from *I. frontalis*, clustered with members of the family Cordycipitaceae, which includes specialized genera of entomopathogenic fungi such as *Lecanicillium*, *Cordyceps*, and *Beauveria* (Fig. 3). In Basidiomycota, eight isolates were assigned to Ustilaginales, two to Tremellales, and two to the Microbotryomycetes *incertae sedis* group. In Mucoromycota, the two isolates were classified under Mucorales. Aside from the Hypocreales isolate c61a, all fungal isolates exhibited high pairwise nucleotide identity with environmental fungi commonly found on vegetation, in the rhizosphere, and in soil, with none associated with human pathogens (Table S2).

### Partitioning of culturable microbial diversity among tick specimens and species

**Figure 4:**
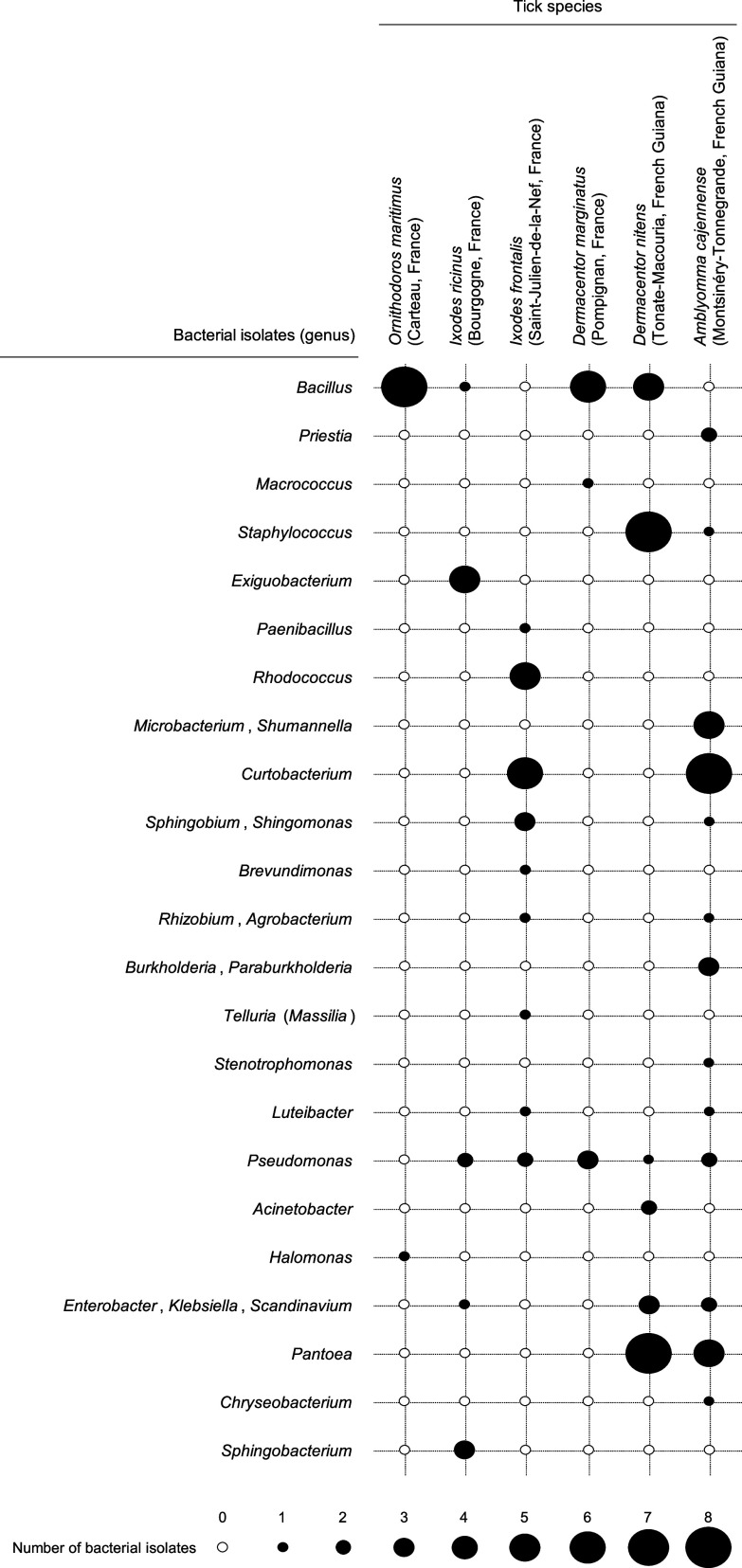
Distribution of isolates per bacterial genus across the six tick species. The size of the black circles represents the number of bacterial isolates.

**Figure 5:**
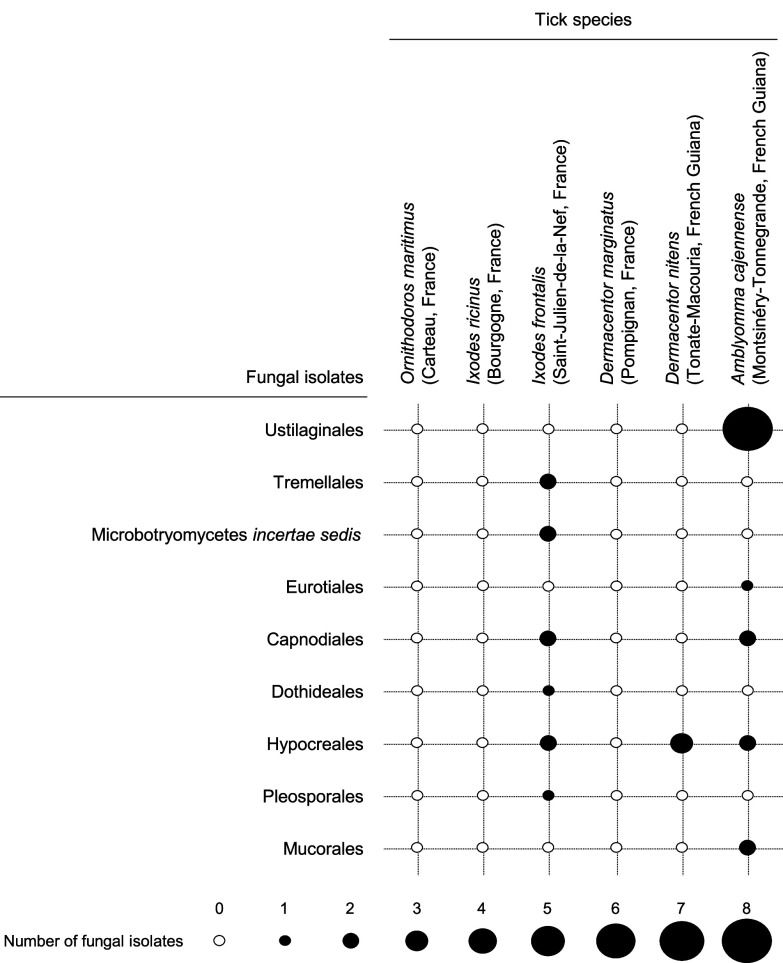
Distribution of isolates per fungal order across the six tick species. The size of the black circles represents the number of bacterial isolates.

Most bacterial genera were frequently isolated from multiple specimens within one or two tick species, yet were rarely or never detected in other species (Fig. 4). As a result, there was significant overall variation in the number of isolates per bacterial genus among the tick species (Fisher’s exact test, *p* = 0.0005). For example, *Pantoea* isolates were recovered from 7 out of 10 *D. nitens* specimens and 3 out of 17 *A. cajennense* specimens, but were absent in other tick species. Additional examples include *Exiguobacterium*, found exclusively in all 4 *I. ricinus* specimens; *Curtobacterium*, isolated only from 8 of the 17 *A. cajennense* specimens and 4 of the 5 *I. frontalis* specimens; *Rhodococcus*, detected solely in 3 of the 5 *I. frontalis* specimens; and *Staphylococcus*, found in 6 of the 10 *D. nitens* specimens and 1 of the 17 *A. cajennense* specimens. Conversely, only a few bacterial genera were isolated from multiple tick species. For instance, *Bacillus* bacteria were found in *O. maritimus* (5 out of 5 specimens), *I. ricinus* (1 of 4 specimens), *D. marginatus* (5 of 6 specimens), and *D. nitens* (4 of 10 specimens). Similarly, *Pseudomonas* were detected in *I. ricinus* (2 of 4 specimens), *I. frontalis* (1 of 5 specimens), *D. marginatus* (2 of 6 specimens), *D. nitens* (1 of 10 specimens), and *A. cajennense* (2 of 17 specimens). A similar pattern was observed for fungi, with significant overall variation in the number of isolates per fungal order among the tick species (*p* = 0.005) (Fig. 5). For example, Ustilaginales and Mucorales isolates were found exclusively in *A. cajennense* (7 and 2 of 17 specimens, respectively). Meanwhile, Hypocreales isolates were detected in *I. frontalis* (2 of 5 specimens), *D. nitens* (2 of 10 specimens), and *A. cajennense* (2 of 17 specimens).

There was no significant variation detected in the number of isolates per bacterial genus or fungal order between tick species from France (*O. maritimus*, *I. ricinus*, *I. frontalis*, and *D. marginatus*) and those from French Guiana (*D. nitens* and *A. cajennense*) (PERMANOVA, bacteria: R^2^ = 0.25, *p* = 0.27; fungi: R^2^ = 0.21, *p* = 0.70). This uniformity was largely driven by bacterial genera such as *Bacillus*, *Curtobacterium*, *Pseudomonas*, and fungal orders like *Capnodiales* and *Hypocreales*, which were present in both European and South American tick species (Figs. 4 and 5). However, two bacterial genera, *Pantoea* and *Staphylococcus*, were only isolated from the two South American tick species (Fig. 4).

## Discussion

The culturable bacteria and fungi we detected in this study reveal considerable microbial diversity with distinct community structures across tick species, aligning with expectations for the tick microbiome [6, 45, 53]. These culturable microbial communities reflect both environmental signatures and tick associations, contributing to the complexity of the tick microbiome. Culturable bacteria showed broader distributions and higher diversity across tick species compared to culturable fungi, although substantial fungal diversity was evident in certain specimens. While few data are currently available on fungi associated with ticks [7, 34, 42], most bacterial genera we isolated (including *Bacillus*, *Acinetobacter*, *Pseudomonas*, *Stenotrophomonas*, *Burkholderia*, and *Chryseobacterium*) were also previously observed in high-throughput metabarcoding analyses [4, 13, 28, 38], demonstrating consistency across different methodological approaches. However, the ability of some of these microbes to be cultured further indicates they are not merely DNA traces from dead environmental microorganisms but rather viable organisms potentially interacting with their tick hosts.

Sequencing of 16S rDNA and 18S rRNA genes evidenced the diversity and host-specificity of culturable microbial communities in ticks. Identical microbial sequences were often characterized in isolates from different specimens of the same tick species, indicating the presence of dominant, species-specific bacterial and fungal strains. For example, bacterial isolates from *I. ricinus* and *D. marginatus* exhibited 100% identical 16S rDNA sequences that were exclusive to their respective species, highlighting a close association between these bacteria and their tick hosts. Similarly, identical fungal sequences were consistently restricted to isolates from the same tick species. This was particularly evident in *A. cajennense*, where seven specimens shared 100% identical 18S rRNA sequences. Importantly, no fungal sequences were shared between different tick species, further supporting the hypothesis of strong host-specific fungal associations. These findings suggest that distinct ecological or evolutionary forces are shaping the relationships between ticks and their bacterial and fungal communities. As a result, this pattern may reflect the ecological and environmental constraints experienced locally by the ticks, or highlight the specialized nature of host-microbe interactions, or both. To confirm this hypothesis, further investigations are needed on a larger number of ticks from diverse localities for each species, as the present study did not encompass such variability. Expanding the geographical and species coverage will be essential to fully understand the ecological and evolutionary drivers of these tick-microbe associations.

Culturable microbial communities varied markedly among the six studied tick species, with certain bacterial genera, such as *Pantoea*, *Bacillus*, and *Curtobacterium*, appearing consistently across tick species. Paradoxically, there were no significant differences between the culturable microbial communities of tick species from France, which live in a temperate continental climate, and those from French Guiana, which inhabit a humid tropical climate. However, there is some evidence indicating that microbial structures are influenced, at least in part, by the ticks’ natural habitats. Interestingly, most isolates found are closely related to generalist bacterial and fungal genera that thrive in diverse terrestrial environments, including soil, rhizospheres, and plant surfaces (Tables S1 and S2). A prime example is the isolation of *Halomonas* bacteria exclusively from *O. maritimus*, a tick species that specifically inhabits marine bird nests in coastal areas. The genus *Halomonas* is known to thrive in saline environments, including estuaries, oceans, and saline lakes [47], and its association with *O. maritimus* reflects the high-salinity habitats this tick species occupies. Other notable isolates include fungal isolates related to the genera *Aureobasidium*, *Cladosporium*, *Fusarium*, *Penicillium*, and *Mucor* (Table S2), which naturally colonize soil and plant surfaces [56, 61] and, in some cases, are major plant pathogens [39]. These findings support the hypothesis that environmental factors shape the observed variability in microbial communities across tick species that occupy distinct ecological niches, a pattern previously observed in tick species [15, 43, 45, 53, 58].

The culturable portion of the tick microbial community may also encompass significant diversity of microorganisms specifically associated with arthropods. Notably, the fungal isolate c61a, primarily obtained from an *I. frontalis* nymph, belongs to the Cordycipitaceae family, a group of entomopathogenic fungi that includes key parasite genera like *Lecanicillium*, *Beauveria*, and *Cordyceps*. These fungi are known to infect a wide range of arthropods by penetrating their cuticle [48, 57], and may therefore potentially affect tick health and survival. Furthermore, entomopathogenic fungi from the genera *Beauveria* and *Metarhizium* are effective in controlling several tick species, including *D. nitens* and *A. cajennense*, although susceptibility varies by tick species, population, and fungal strain [11, 26, 52]. Another notable example includes members of the *Pantoea* genus, which were isolated from *D. nitens* and *A. cajennense* in this study and have also previously been reported in other tick species, such as *I. ricinus* [59]. *Pantoea* bacteria are emerging as widespread microorganisms, known for their adaptability to diverse environments and hosts, including plants, insects, and humans [60]. Recently, mutualistic *Pantoea* gut symbionts have been identified in diverse arthropods, such as pentatomid stinkbugs, where the bacteria supply nutrients unavailable from plant sap food sources [22, 32]. However, their role in ticks remains unknown. Similarly, other bacterial genera, such as *Chryseobacterium*, have previously been detected in the digestive tracts of mosquitoes, cockroaches, and millipedes [17, 35, 36] and may be important partners of tick midguts. Further research should thus investigate whether these bacteria engage in specialized relationships with ticks and whether they influence tick health, nutrition, or vector competence. Notably, the role of arthropod-associated microbes may be even more significant than this study suggests. Their true diversity is likely underestimated, as our generalist culture medium may not support the growth of many bacteria, especially those highly specialized for the internal biology of ticks. A prime example is intracellular bacteria, such as nutritional symbionts, which are abundant in ticks yet remain unculturable under standard laboratory conditions [21, 29].

Although informative, the use of the 16S rRNA and 18S rRNA gene sequences as exclusive genetic markers for bacterial and fungal isolates, respectively, represents a significant limitation in the interpretation of these datasets, as it restricts the ability to accurately identify and characterize the microbial species present in the tick samples. The taxonomic resolution of the 16S rRNA gene is limited, particularly for distinguishing closely related species: many bacteria share highly similar 16S rRNA sequences, leading to misidentifications, especially when species are genetically similar but phenotypically distinct [23, 33]. Similarly, the 18S rRNA gene, widely used for fungal identification, is highly conserved within fungal groups, limiting its ability to differentiate closely related species or resolve cryptic species. The lack of variability in this gene often results in broad-level identification, which is insufficient for precise species-level classification, as observed in this study. Consequently, the reduced taxonomic resolution of the 16S rRNA and 18S rRNA gene sequences may mask a substantial portion of the microbial community composition, interactions, and ecological roles. This may be particularly problematic for microbial taxa with conserved genetic regions that exhibit subtle ecological or functional differences, as for *Pantoea* bacteria [60]. Sequencing more variable genetic markers would provide higher taxonomic resolution, allowing for more accurate microbial typing and uncovering hidden diversity overlooked by 16S rRNA and 18S rRNA genes. This approach could reveal finer-scale ecological or functional distinctions, offering deeper insights into tick-associated microbial communities.

In conclusion, the microbiome structure formed by culturable bacteria and fungi reflects both environmental and host tick influences and may play a potential role in tick biology and ecology. While the primary role of these microbes in ticks remains to be elucidated, the identification of taxa with known arthropod associations provides a foundation for future research on tick-microbe interactions. Fungi associated with ticks remain a significantly understudied compartment of the tick microbiome, despite their potential ecological and pathogenic roles. Future studies employing both culture-dependent and culture-independent methods could better define the functional roles of these microbes in tick health, pathogen resistance, and the potential effects on host-vector dynamics.

## Supplementary material

The supplementary material for this article can be found at https://www.parasite-journal.org/10.1051/parasite/2025013/olm. *Table S1*: Bacterial isolates of ticks and best nucleotide identities with 16S rRNA gene sequences obtained in this study and sequences available in GenBank identified through Basic Local Alignment Search Tool (BLAST; https://blast.ncbi.nlm.nih.gov/Blast.cgi) searches (Last update: 10/10/2024). *Table S2*: Fungal isolates of ticks and best nucleotide identities with 18S rRNA gene sequences obtained in this study and sequences available in GenBank identified through Basic Local Alignment Search Tool (BLAST; https://blast.ncbi.nlm.nih.gov/Blast.cgi) searches (Last update: 10/10/2024).
