# Supplementary material for: Culturable bacteria and fungi in Ixodes, Dermacentor, Amblyomma and Ornithodoros ticks
Source: Parasite. 2025 Mar 25;32:20. doi: 10.1051/parasite/2025013 (PMC11936413; doi:10.1051/parasite/2025013)
Supplement: Supplementary file 1 — Table S1: Bacterial isolates of ticks and best nucleotide identities with 16S rRNA gene sequences obtained in this study and sequences available in GenBank identified through Basic Local Alignment Search Tool (BLAST; https://blast.ncbi.nlm.nih.gov/Blast.cgi) searches (Last update: 10/10/2024). [file parasite-32-20-s1.pdf]

**Table S1.** Bacterial isolates of ticks and best nucleotide identities with 16S rRNA gene sequences obtained in this study and sequences available in GenBank identified through Basic Local Alignment Search Tool (BLAST; https://blast.ncbi.nlm.nih.gov/Blast.cgi) searches (Last update: 10/10/2024).

| Tick species | Bacterial isolate id | Best match in GenBank | | | | | | |
|---|---|---|---|---|---|---|---|---|
| | | Scientific name | Query cover | E-value | % identity | Accession number | Country | Isolate source |
| *Ornithodoros maritimus* | C51a | *Bacillus* sp. | 100% | 0.0 | 99.72% | OR392979.1 | unknown | Unknown |
| *Ornithodoros maritimus* | C52a | *Bacillus* sp. | 100% | 0.0 | 99.72% | OR392979.1 | unknown | Unknown |
| *Ornithodoros maritimus* | C52b | *Bacillus* sp. | 100% | 0.0 | 100.00% | OR835281.1 | unknown | Unknown |
| *Ornithodoros maritimus* | C52c | *Bacillus* sp. | 100% | 0.0 | 99.72% | OR392979.1 | unknown | Unknown |
| *Ornithodoros maritimus* | C53b | *Bacillus* sp. | 100% | 0.0 | 100.00% | OR835281.1 | unknown | Unknown |
| *Ornithodoros maritimus* | C54a | *Bacillus* sp. | 100% | 0.0 | 100.00% | OR835281.1 | unknown | Unknown |
| *Ornithodoros maritimus* | C54b | *Halomonas* sp. | 100% | 0.0 | 99.71% | CP020562.1 | China | Textile industrial wastewaters |
| *Ornithodoros maritimus* | C55a | *Bacillus* sp. | 100% | 0.0 | 100.00% | OR835281.1 | unknown | Unknown |
| *Ornithodoros maritimus* | C55c | *Bacillus* sp. | 100% | 0.0 | 99.72% | OR392979.1 | unknown | Unknown |
| *Amblyomma cajennense* | C21a | *Pantoea dispersa* | 100% | 0.0 | 100.00% | MN067798.1 | Sri Lanka | Mid gut *Phlebotomus argentipes* |
| *Amblyomma cajennense* | C21b | *Pantoea dispersa* | 100% | 0.0 | 100.00% | MN067798.1 | Sri Lanka | Mid gut *Phlebotomus argentipes* |
| *Amblyomma cajennense* | C22b | *Curtobacterium luteum* | 100% | 0.0 | 100.00% | MN889291.1 | India | Leaf |
| *Amblyomma cajennense* | C22d | *Curtobacterium luteum* | 100% | 0.0 | 100.00% | MN889291.1 | India | Leaf |
| *Amblyomma cajennense* | C23b | *Curtobacterium* sp. | 100% | 0.0 | 100.00% | MK704290.1 | unknown | Unknown |
| *Amblyomma cajennense* | C23d | *Luteibacter yeojuensis* | 98% | 0.0 | 99.86% | OP811701.1 | France | *Vanilla planifolia* |
| *Amblyomma cajennense* | C24b | *Curtobacterium* sp. | 100% | 0.0 | 100.00% | MK704290.1 | unknown | Unknown |
| *Amblyomma cajennense* | C25 | *Scandinavium* sp. | 100% | 0.0 | 99.57% | PP412028.2 | South Korea | Lettuce |
| *Amblyomma cajennense* | C26a | *Pantoea dispersa* | 100% | 0.0 | 100.00% | MN067798.1 | Sri Lanka | Mid gut phlebotomus argentipes |
| *Amblyomma cajennense* | C26b | *Pantoea soli* | 100% | 0.0 | 99.93% | CP032702.1 | Singapore | Soil |
| *Amblyomma cajennense* | C28a | *Microbacterium* sp. | 100% | 0.0 | 100.00% | CP151633.1 | Switzerland | Zea mays |
| *Amblyomma cajennense* | C28b | *Stenotrophomonas* sp. | 100% | 0.0 | 100.00% | MK398096.1 | Poland | *Salicornia europaea* |
| *Amblyomma cajennense* | C29a | *Bacillus* sp. | 100% | 0.0 | 100.00% | PP892114.1 | China | Unknown |
| *Amblyomma cajennense* | C29b | *Pseudomonas* sp. | 100% | 0.0 | 99.71% | OP132331.1 | USA | Organic rice seeds |
| *Amblyomma cajennense* | C29c | *Microbacterium testaceum* | 100% | 0.0 | 100.00% | JQ660317.1 | Singapore | Plant tissue |
| *Amblyomma cajennense* | C29d | *Curtobacterium luteum* | 100% | 0.0 | 99.86% | MT367778.1 | India | Leaf |
| *Amblyomma cajennense* | C30 | *Enterobacter* sp. | 100% | 0.0 | 99.86% | MW375575.1 | Malaysia | Forest soils |
| *Amblyomma cajennense* | C31b | *Staphylococcus cohnii* | 100% | 0.0 | 100.00% | MT235755.1 | China | Nasal swab |
| *Amblyomma cajennense* | C31c | *Curtobacterium* sp. | 100% | 0.0 | 100.00% | MT367788.1 | India | Leaf (*Oryza sativa*) |
| *Amblyomma cajennense* | C33 | *Burkholderia gladioli* | 100% | 0.0 | 100.00% | MT626030.1 | unknown | Rice |
| *Amblyomma cajennense* | C35b | *Microbacterium trichothecenolyticum* | 100% | 0.0 | 99.93% | MT275627.1 | Sri Lanka | Midgut of Aedes albopictus adults |
| *Amblyomma cajennense* | C35c | *Schumannella luteola* | 100% | 0.0 | 99.79% | OK562840.1 | unknown | Unknown |
| *Amblyomma cajennense* | C36a | *Pseudomonas* sp. | 99% | 0.0 | 100.00% | KJ184947.1 | unknown | Sugarcane |
| *Amblyomma cajennense* | C36c | *Agrobacterium cavarae* | 100% | 0.0 | 100.00% | MK940276.1 | Spain | Maize roots |
| *Amblyomma cajennense* | C37a | *Bacillus sp.* | 100% | 0.0 | 100.00% | MT065750.1 | unknown | Unknown |
| *Amblyomma cajennense* | C37c | *Burkholderia tropica* | 100% | 0.0 | 99.79% | KT390912.1 | Brazil | Nodule |
| *Amblyomma cajennense* | C37d | *Curtobacterium luteum* | 100% | 0.0 | 100.00% | MN889291.1 | India | Leaf |
| *Amblyomma cajennense* | C37e | *Burkholderia tropica* | 100% | 0.0 | 99.79% | KT390912.1 | Brazil | Plant nodule |
| *Amblyomma cajennense* | C38b | *Chryseobacterium* sp. | 99% | 0.0 | 99.15% | OR529363.1 | unknown | Unknown |
| *Amblyomma cajennense* | C38d | *Curtobacterium flaccumfaciens* | 100% | 0.0 | 99.93% | CP041259.1 | Turkey | Phaseolus vulgaris |
| *Amblyomma cajennense* | C38e | *Sphingobium rhizovicinum* | 99% | 0.0 | 100% | MW052572.1 | China | Air |
| *Amblyomma cajennense* | C39 | *Pantoea dispersa* | 100% | 0.0 | 100.00% | MT275631.1 | Sri Lanka | Midgut of *Aedes albopictus* adults |
| *Dermacentor nitens* | C1a | *Pantoea eucrina* | 100% | 0.0 | 99.72% | CP083448.1 | China | Cucumber Rhizosphere |
| *Dermacentor nitens* | C1b | *Staphylococcus* sp. | 100% | 0.0 | 99.86% | OQ255846.1 | Turkey | Lake |
| *Dermacentor nitens* | C2a | *Pantoea soli* | 100% | 0.0 | 99.86% | CP032702.1 | Singapore | Soil |
| *Dermacentor nitens* | C2b | *Staphylococcus* sp. | 100% | 0.0 | 99.93% | OQ255846.1 | Turkey | Lake |
| *Dermacentor nitens* | C3a | *Pantoea stewartii* | 100% | 0.0 | 99.93% | CP099540.1 | China | Rice (*Oryza sativa*) |
| *Dermacentor nitens* | C3b | *Staphylococcus* sp. | 100% | 0.0 | 99.93% | OQ255846.1 | Turkey | Lake |

| | | | | | | | | |
|---|---|---|---|---|---|---|---|---|
| *Dermacentor nitens* | C4a | *Bacillus cereus* | 100% | 0.0 | 100.00% | MT642947.1 | Malaysia | sediment |
| *Dermacentor nitens* | C4b | *Klebsiella grimontii* | 100% | 0.0 | 100.00% | MT538677.1 | unknown | Unknown |
| *Dermacentor nitens* | C5a | *Bacillus cereus* | 100% | 0.0 | 100.00% | MT642947.1 | Malaysia | Sediment |
| *Dermacentor nitens* | C5b | *Enterobacter hormaechei* | 98% | 0.0 | 100.00% | CP045611.1 | Lebanon | Unknown |
| *Dermacentor nitens* | C6a | *Pantoea eucrina* | 100% | 0.0 | 99.86% | MF135172.1 | unknown | Rice |
| *Dermacentor nitens* | C6b | *Pantoea eucrina* | 100% | 0.0 | 99.86% | MF135172.1 | unknown | Rice |
| *Dermacentor nitens* | C6d | *Staphylococcus* sp. | 100% | 0.0 | 100.00% | OQ255846.1 | Turkey | Lake |
| *Dermacentor nitens* | C6e | *Staphylococcus* sp. | 100% | 0.0 | 99.86% | OQ255846.1 | Turkey | Lake |
| *Dermacentor nitens* | C6f | *Pantoea eucrina* | 100% | 0.0 | 100.00% | MT367823.1 | India | Leaf |
| *Dermacentor nitens* | C6g | *Staphylococcus* sp. | 100% | 0.0 | 99.86% | OQ255846.1 | Turkey | Lake |
| *Dermacentor nitens* | C8a | *Acinetobacter nosocomialis* | 100% | 0.0 | 100.00% | CP010368.1 | unknown | Unknown |
| *Dermacentor nitens* | C8b | *Bacillus cereus* | 100% | 0.0 | 100.00% | MT642947.1 | Malaysia | Sediment |
| *Dermacentor nitens* | C8c | *Bacillus cereus* | 100% | 0.0 | 100.00% | MT642947.1 | Malaysia | Sediment |
| *Dermacentor nitens* | C10c | *Enterobacter ludwigii* | 100% | 0.0 | 100.00% | MT613372.1 | China | Plant endophyte |
| *Dermacentor nitens* | C10d | *Pantoea eucrina* | 100% | 0.0 | 99.65% | CP083448.1 | China | Cucumber rhizosphere |
| *Dermacentor nitens* | C11b | *Bacillus cereus* | 100% | 0.0 | 100.00% | MT642947.1 | Malaysia | Sediment |
| *Dermacentor nitens* | C11c | *Pantoea eucrina* | 100% | 0.0 | 99.86% | MF135172.1 | unknown | Rice |
| *Dermacentor nitens* | C11d | *Acinetobacter septicus* | 100% | 0.0 | 99.93% | MN725744.1 | Colombia | Ovaries *Aedes aegypti* |
| *Dermacentor nitens* | C15a | *Staphylococcus* sp. | 100% | 0.0 | 100.00% | OQ255846.1 | Turkey | Lake |
| *Dermacentor nitens* | C15b | *Pseudomonas monteilii* | 100% | 0.0 | 100.00% | MT605299.1 | unknown | Unknown |
| *Dermacentor nitens* | C15c | *Staphylococcus schleiferi* | 100% | 0.0 | 100.00% | MF678906.1 | unknown | Jugular catheter |
| *Dermacentor marginatus* | C41 | *Bacillus thuringiensis* | 100% | 0.0 | 98.46% | CP053972.1 | USA | Unknown |
| *Dermacentor marginatus* | C42 | *Bacillus thuringiensis* | 100% | 0.0 | 98.46% | CP053972.1 | USA | Unknown |
| *Dermacentor marginatus* | C43a | *Pseudomonas alcaligenes* | 100% | 0.0 | 96.45% | MK719951.1 | China | Unknown |
| *Dermacentor marginatus* | C43b | *Macrococcus equipercicus* | 100% | 0.0 | 100.00% | CP073809.1 | Switzerland | Ear swab |
| *Dermacentor marginatus* | C44 | *Bacillus thuringiensis* | 100% | 0.0 | 98.46% | CP053972.1 | USA | Unknown |
| *Dermacentor marginatus* | C45 | *Bacillus thuringiensis* | 99% | 0.0 | 99.04% | CP050183.1 | Canada | Unknown |
| *Dermacentor marginatus* | C46a | *Pseudomonas* sp. | 100% | 0.0 | 99.36% | LC230077.1 | Japan | Soil |
| *Dermacentor marginatus* | C46b | *Bacillus thuringiensis* | 100% | 0.0 | 98.46% | CP053972.1 | USA | Unknown |
| *Dermacentor marginatus* | C46c | *Pseudomonas alcaligenes* | 100% | 0.0 | 96.45% | MK719951.1 | China | Unknown |
| *Dermacentor marginatus* | C46d | *Bacillus thuringiensis* | 99% | 0.0 | 99.04% | CP050183.1 | Canada | Unknown |
| *Ioxdes frontalis* | C58b | *Massilia aurea* | 100% | 0.0 | 100.00% | MK720412.1 | China | Taklamakan desert |
| *Ioxdes frontalis* | C58c | *Curtobacterium citreum* | 100% | 0.0 | 97.65% | MF319766.1 | India | Cauliflower leaf |
| *Ioxdes frontalis* | C58e | *Curtobacterium citreum* | 100% | 0.0 | 97.65% | MF319766.1 | India | Cauliflower leaf |
| *Ioxdes frontalis* | C59a | *Pseudomonas* sp. | 100% | 0.0 | 99.40% | MK559936.1 | Spain | *Quercus pyrenaica* rhizosphere |
| *Ioxdes frontalis* | C59b | *Pseudomonas* sp. | 100% | 0.0 | 99.40% | MK559936.1 | Spain | *Quercus pyrenaica* rhizosphere |
| *Ioxdes frontalis* | C59c | *Rhodococcus corynebacterioides* | 100% | 0.0 | 99.78% | MN826595.1 | unknown | Unknown |
| *Ioxdes frontalis* | C60b | *Paenibacillus* sp. | 100% | 0.0 | 99.86% | CP150238.1 | unknown | Milk |
| *Ioxdes frontalis* | C60c | *Curtobacterium citreum* | 100% | 0.0 | 97.65% | MF319766.1 | India | Cauliflower leaf |
| *Ioxdes frontalis* | C60d | *Luteibacter rhizovicinus* | 100% | 0.0 | 100.00% | MT008462.1 | Poland | Soil |
| *Ioxdes frontalis* | C61b | *Rhodococcus* sp. | 100% | 0.0 | 99.34% | MT012186.1 | Tunisia | Unknown |
| *Ioxdes frontalis* | C61c | *Sphingomonas* sp. | 100% | 0.0 | 99.70% | MN989146.1 | unknown | Compound leaves of *Fraxinus excelsior* |
| *Ioxdes frontalis* | C61d | *Curtobacterium flaccumfaciens* | 100% | 0.0 | 99.56% | CP041259.1 | Turkey | Unknown |
| *Ioxdes frontalis* | C62a | *Curtobacterium citreum* | 100% | 0.0 | 97.65% | MF319766.1 | India | Cauliflower leaf |
| *Ioxdes frontalis* | C62d | *Rhodococcus yunnanensis* | 99% | 0.0 | 99.95% | KY783360.1 | Svalbard | Sediment core from Kongsfzorde |
| *Ioxdes frontalis* | C62e | *Sphingomonadaceae bacterium* | 99% | 0.0 | 99.41% | CP099621.1 | France | Leaf |
| *Ioxdes frontalis* | C62g | *Rhodococcus* sp. | 100% | 0.0 | 99.49% | CP032762.1 | Russia | Podzolic fallow soil |
| *Ioxdes frontalis* | C62h | *Rhodococcus* sp. | 100% | 0.0 | 99.49% | CP032762.1 | Russia | Podzolic fallow soil |
| *Ioxdes frontalis* | C62i | *Sphingomonas* sp. | 100% | 0.0 | 99.70% | MN989146.1 | unknown | Compound leaves of *Fraxinus excelsior* |
| *Ioxdes frontalis* | C62j | *Brevundimonas intermedia* | 100% | 0.0 | 100.00% | MW433644.1 | unknown | Unknown |
| *Ioxdes frontalis* | C62l | *Curtobacterium citreum* | 100% | 0.0 | 97.65% | MF319766.1 | India | Cauliflower leaf |
| *Ioxdes frontalis* | C62m | *Pararhizobium qamdonense* | 100% | 0.0 | 99.21% | CP119566.1 | China | Soil |
| *Ixodes ricinus* | C47a | *Bacillus cereus* | 99% | 0.0 | 100.00% | CP128166.1 | Denmark | Grassland soil |
| *Ixodes ricinus* | C47b | *Exiguobacterium* sp. | 99% | 0.0 | 99.79% | CP053557.1 | Spain | Solar panel array |
| *Ixodes ricinus* | C47c | *Pseudomonas* sp. | 100% | 0.0 | 99.36% | LC230077.1 | Japan | Soil |
| *Ixodes ricinus* | C47d | *Sphingobacterium kitahiroshimense* | 100% | 0.0 | 99.78% | MK402063.1 | South Korea | Unknown |
| *Ixodes ricinus* | C48a | *Exiguobacterium* sp. | 99% | 0.0 | 99.79% | CP053557.1 | Spain | Solar panel array |
| *Ixodes ricinus* | C48d | *Sphingobacterium kitahiroshimense* | 100% | 0.0 | 99.93% | MK402063.1 | South Korea | Unknown |

| | | | | | | | | |
|---|---|---|---|---|---|---|---|---|
| *Ixodes ricinus* | C48e | *Exiguobacterium* sp. | 99% | 0.0 | 99.79% | CP053557.1 | Spain | Solar panel array |
| *Ixodes ricinus* | C49a | *Exiguobacterium* sp. | 99% | 0.0 | 99.79% | CP053557.1 | Spain | Solar panel array |
| *Ixodes ricinus* | C49b | *Pseudomonas* sp. | 100% | 0.0 | 99.36% | LC230077.1 | Japan | Soil |
| *Ixodes ricinus* | C50a | *Exiguobacterium* sp. | 99% | 0.0 | 99.79% | CP053557.1 | Spain | Solar panel array |
| *Ixodes ricinus* | C50b | *Sphingobacterium kitahiroshimense* | 100% | 0.0 | 99.93% | MK402063.1 | South Korea | Unknown |
| *Ixodes ricinus* | C50c | *Enterobacter* sp. | 100% | 0.0 | 99.71% | CP082269.1 | China | Root rhizomes |
