# Supplementary material for: Culturable bacteria and fungi in Ixodes, Dermacentor, Amblyomma and Ornithodoros ticks
Source: Parasite. 2025 Mar 25;32:20. doi: 10.1051/parasite/2025013 (PMC11936413; doi:10.1051/parasite/2025013)
Supplement: Supplementary file 2 — Table S2: Fungal isolates of ticks and best nucleotide identities with 18S rRNA gene sequences obtained in this study and sequences available in GenBank identified through Basic Local Alignment Search Tool (BLAST; https://blast.ncbi.nlm.nih.gov/Blast.cgi) searches (Last update: 10/10/2024). [file parasite-32-20-s2.pdf]

Table S2. Fungal isolates of ticks and best nucleotide identities with 18S rRNA gene sequences obtained in this study and sequences available in GenBank identified through Basic Local Alignment Search Tool (BLAST; https://blast.ncbi.nlm.nih.gov/Blast.cgi) searches (Last update: 10/10/2024).

Tick species	Fungal isolate id	Best match in GenBank						
		Scientific name	Query cover	E-value	% identity	Accession	Country	Isolate source
Ixodes frontalis	c58a	*Cladosporium cladosporioides*	99%	0.0	100.00%	OR243761.1	Unknown	Unknown
Ixodes frontalis	c58d	*Hannaella oryzae*	100%	0.0	99.44%	NG_063522.1	Unknown	Culture from holotype of *Bullera oryzae*
Ixodes frontalis	c60a	*Cladosporium cladosporioides*	99%	0.0	100.00%	OR243761.1	Unknown	Unknown
Ixodes frontalis	c60e	*Bulleribasidium wuzhishanense*	100%	0.0	99.44%	GQ438830.1	Unknown	Unknown
Ixodes frontalis	c61a	*Lecanicillium psalliotae*	100%	0.0	99.72%	KM013704.1	China	Asian citrus psyllid (*Diaphorina citri*)
Ixodes frontalis	c62b	*Pseudohyphozyma hydrangeae*	100%	0.0	99.72%	MK050443.1	Unknown	Unknown
Ixodes frontalis	c62c	*Aureobasidium sp.*	100%	0.0	100.00%	MN268776.1	Unknown	Unknown
Ixodes frontalis	c62f	*Parastagonospora nodorum*	100%	0.0	99.44%	CP039677.1	Unknown	Wild grass leaf tissue
Ixodes frontalis	c62k	*Pseudohyphozyma hydrangeae*	100%	0.0	99.72%	MK050443.1	Unknown	Unknown
Dermacentor nitens	c1c	*Fusarium oxysporum*	100%	0.0	100.00%	CP052041.1	Unknown	Soil
Dermacentor nitens	c2c	*Fusarium oxysporum*	100%	0.0	100.00%	CP052041.1	Unknown	Soil
Dermacentor nitens	c6c	*Fusarium fujikuroi*	100%	0.0	100.00%	CP023090.1	Italy	Rice plant
Amblyomma cajennense	c22a	*Penicillium decumbens*	100%	0.0	100.00%	KX553859.1	Unknown	Rice root soil
Amblyomma cajennense	c22c	*Moesziomyces antarcticus*	100%	0.0	100.00%	CP080180.1	South Korea	Brown rice
Amblyomma cajennense	c23a	*Mucor* sp.	82%	0.0	100.00%	MT523893.1	Benin	Unknown
Amblyomma cajennense	c23c	*Moesziomyces antarcticus*	100%	0.0	100.00%	CP080180.1	South Korea	Brown rice
Amblyomma cajennense	c24a	*Mucor* sp.	100%	0.0	97.81%	MT280228.1	Unknown	Unknown
Amblyomma cajennense	c24c	*Moesziomyces antarcticus*	100%	0.0	100.00%	CP080180.1	South Korea	Brown rice
Amblyomma cajennense	c27a	*Cladosporium cladosporioides*	100%	0.0	100.00%	OR243761.1	Unknown	Unknown
Amblyomma cajennense	c27b	*Moesziomyces antarcticus*	100%	0.0	100.00%	CP080180.1	South Korea	Brown rice
Amblyomma cajennense	c27c	*Moesziomyces antarcticus*	100%	0.0	100.00%	CP080180.1	South Korea	Brown rice
Amblyomma cajennense	c31a	*Myrothecium leucotrichum*	100%	0.0	99.72%	AJ301992.1	Unknown	Unknown
Amblyomma cajennense	c35a	*Moesziomyces antarcticus*	100%	0.0	100.00%	CP080180.1	South Korea	Brown rice
Amblyomma cajennense	c36b	*Cladosporium cladosporioides*	100%	0.0	100.00%	OR243761.1	Unknown	Unknown
Amblyomma cajennense	c37b	*Moesziomyces antarcticus*	100%	0.0	100.00%	CP080180.1	South Korea	Brown rice
Amblyomma cajennense	c38a	*Chlamydocillium curvulum*	100%	0.0	99.44%	NG_062811.1	Unknown	Unknown
Amblyomma cajennense	c38c	*Moesziomyces antarcticus*	100%	0.0	100.00%	CP080180.1	South Korea	Brown rice
